# Tobacco Product Use and Cessation Indicators Among Adults — United States, 2018

**DOI:** 10.15585/mmwr.mm6845a2

**Published:** 2019-11-15

**Authors:** MeLisa R. Creamer, Teresa W. Wang, Stephen Babb, Karen A. Cullen, Hannah Day, Gordon Willis, Ahmed Jamal, Linda Neff

**Affiliations:** ^1^Office on Smoking and Health, National Center for Chronic Disease Prevention and Health Promotion, CDC; ^2^Center for Tobacco Products, Food and Drug Administration, Silver Spring, Maryland; ^3^Tobacco Control Research Branch, National Cancer Institute, National Institutes of Health, Bethesda, Maryland.

Cigarette smoking is the leading cause of preventable disease and death in the United States (*1*). The prevalence of adult cigarette smoking has declined in recent years to 14.0% in 2017 (*2*). However, an array of new tobacco products, including e-cigarettes, has entered the U.S. market (*3*). To assess recent national estimates of tobacco product use among U.S. adults aged ≥18 years, CDC, the Food and Drug Administration (FDA), and the National Cancer Institute analyzed data from the 2018 National Health Interview Survey (NHIS). In 2018, an estimated 49.1 million U.S. adults (19.7%) reported currently using any tobacco product, including cigarettes (13.7%), cigars (3.9%), e-cigarettes (3.2%), smokeless tobacco (2.4%), and pipes* (1.0%). Most tobacco product users (83.8%) reported using combustible products (cigarettes, cigars, or pipes), and 18.8% reported using two or more tobacco products. The prevalence of any current tobacco product use was higher in males; adults aged ≤65 years; non-Hispanic American Indian/Alaska Natives; those with a General Educational Development certificate (GED); those with an annual household income <$35,000; lesbian, gay, or bisexual adults; uninsured adults; those with a disability or limitation; and those with serious psychological distress. The prevalence of e-cigarette and smokeless tobacco use increased during 2017–2018. During 2009–2018, there were significant increases in all three cigarette cessation indicators (quit attempts, recent cessation, and quit ratio). Implementing comprehensive population-based interventions in coordination with regulation of the manufacturing, marketing, and distribution of all tobacco products can reduce tobacco-related disease and death in the United States (*1*,*4*).

NHIS is an annual, nationally representative, household survey of the noninstitutionalized U.S. civilian population.^†^ The 2018 NHIS Sample Adult component included 25,417 adults aged ≥18 years; the response rate was 53.1% (*5*). Data were weighted to provide nationally representative estimates, adjusting for differences in selection probability and nonresponse. Use of five tobacco products was assessed: cigarettes, cigars (cigars, cigarillos, or filtered little cigars), pipes (regular pipes, water pipes, or hookahs), e-cigarettes, and smokeless tobacco (chewing tobacco, snuff, dip, snus, or dissolvable tobacco). Current cigarette smokers reported having smoked ≥100 cigarettes during their lifetime and smoked every day or some days at the time of survey. Current users of all other tobacco products reported using these products every day or some days at the time of survey. Prevalence estimates for current use of any tobacco product, any combustible tobacco product, and ≥2 tobacco products^§^ were calculated. Estimates were calculated overall and separately by sex, age, race/ethnicity, U.S. Census region, education (for adults aged ≥25 years), marital status, annual household income, sexual orientation, health insurance coverage, disability, and presence of serious psychological distress. T-tests were performed to assess overall differences in tobacco product use from 2017 to 2018.^¶^ Daily and nondaily use of each product was assessed. Three cigarette smoking cessation indicators were assessed: past-year quit attempts,** recent successful cessation,^††^ and quit ratio.^§§^ Linear and nonlinear (quadratic) trends were assessed for each cigarette smoking cessation indicator during 2009–2018. Statistical significance was defined as p<0.05 for differences and trends. SAS-Callable SUDAAN software (version 11.0.3; Research Triangle Institute) was used to conduct all analyses; all analyses were weighted and accounted for the complex survey design.

Among U.S. adults in 2018, 19.7% (estimated 49.1 million) currently used any tobacco product, 16.5% (41.2 million; 83.8% of current tobacco users) used any combustible tobacco product, and 3.7% (9.3 million; 18.8% of current tobacco users) used ≥2 tobacco products (Table). Cigarettes were the most commonly used tobacco product (13.7%; 34.2 million). Prevalence estimates of use of the other tobacco products in 2018 were as follows: cigars (3.9%; 9.6 million); e-cigarettes (3.2%; 8.1 million); smokeless tobacco (2.4%; 5.9 million); and pipes (1.0%; 2.6 million). During 2017–2018, the prevalence of e-cigarette use increased from 2.8% to 3.2% (p = 0.029), and the prevalence of smokeless tobacco use increased from 2.1% to 2.4% (p = 0.047). No significant changes occurred in the use of the other tobacco products included in this study. Among current tobacco product users, daily use was reported by 74.6% of cigarette smokers, 59.1% of smokeless tobacco users, 42.6% of e-cigarette users, and 15.8% of cigar smokers (Figure 1).^¶¶^

**TABLE:** Percentage of persons aged ≥18 years who reported tobacco product use “every day” or “some days,” by tobacco product and selected characteristics — National Health Interview Survey, United States, 2018

Demographic	% (95% CI)							
	Any tobacco product*	Any combustible product^†^	Cigarettes^§^	Cigars/ Cigarillos/ Filtered little cigars^¶^	Pipe/ Water pipe/ Hookah**	E-cigarettes^††^	Smokeless tobacco^§§^	≥2 Tobacco products^¶¶^
**Overall**	**19.7 (19.0–20.4)**	**16.5 (15.9–17.2)**	**13.7 (13.1–14.3)**	**3.9 (3.5–4.2)**	**1.0 (0.9–1.2)**	**3.2 (3.0–3.5)**	**2.4 (2.1–2.6)**	**3.7 (3.4–4.0)**
**Sex**								
Men	25.8 (24.7–26.9)	20.6 (19.6–21.5)	15.6 (14.8–16.5)	6.8 (6.2–7.4)	1.5 (1.3–1.8)	4.3 (3.8–4.8)	4.7 (4.2–5.1)	5.9 (5.3–6.4)
Women	14.1 (13.3–14.9)	12.8 (12.0–13.5)	12.0 (11.2–12.7)	1.1 (0.8–1.3)	0.6 (0.4–0.7)	2.3 (2.0–2.6)	—***	1.7 (1.5–2.0)
**Age group (yrs)**								
18–24	17.1 (14.8–19.3)	11.2 (9.3–13.1)	7.8 (6.2–9.4)	4.1 (2.9–5.3)	—	7.6 (6.1–9.1)	—	4.1 (3.0–5.2)
25–44	23.8 (22.5–25.0)	20.0 (18.9–21.2)	16.5 (15.4–17.6)	5.0 (4.4–5.6)	1.5 (1.1–1.8)	4.3 (3.7–4.8)	3.2 (2.7–3.6)	5.5 (4.9–6.1)
45–64	21.3 (20.2–22.4)	18.7 (17.6–19.7)	16.3 (15.3–17.3)	3.7 (3.2–4.2)	0.6 (0.4–0.8)	2.1 (1.8–2.5)	2.4 (2.0–2.8)	3.3 (2.8–3.7)
≥65	11.9 (11.0–12.8)	10.3 (9.5–11.1)	8.4 (7.7–9.2)	2.1 (1.7–2.5)	—	0.8 (0.6–1.1)	1.4 (1.0–1.7)	1.3 (1.0–1.6)
**Race/Ethnicity^†††^**								
White	21.9 (21.1–22.8)	17.9 (17.1–18.6)	15.0 (14.3–15.7)	4.1 (3.7–4.5)	1.0 (0.8–1.2)	3.7 (3.3–4.1)	3.3 (2.9–3.6)	4.2 (3.8–4.6)
Black	19.3 (17.3–21.3)	18.2 (16.3–20.1)	14.6 (12.8–16.3)	4.9 (3.8–5.9)	—	—	—	3.5 (2.7–4.3)
Asian	10.0 (8.0–12.0)	8.2 (6.3–10.0)	7.1 (5.2–8.9)	—	—	—	—	—
AI/AN	32.3 (19.1–45.5)	25.2 (14.4–35.9)	22.6 (12.0–33.3)	—	—	—	—	—
Hispanic	13.8 (12.2–15.4)	12.3 (10.8–13.8)	9.8 (8.4–11.2)	2.8 (2.0–3.5)	—	2.5 (1.8–3.3)	—	2.2 (1.4–3.0)
Multiracial	25.4 (19.8–30.9)	21.3 (16.2–26.3)	19.1 (14.3–24.0)	—	—	—	—	—
**U.S. Census region^§§§^**								
Northeast	17.5 (15.8–19.1)	15.7 (14.2–17.2)	12.5 (11.1–13.8)	4.5 (3.6–5.4)	–	2.2 (1.7–2.7)	1.3 (0.8–1.8)	3.4 (2.5–4.2)
Midwest	23.6 (22.0–25.1)	19.7 (18.3–21.1)	16.2 (15.0–17.5)	4.8 (3.9–5.6)	1.1 (0.7–1.4)	4.0 (3.3–4.6)	3.0 (2.4–3.5)	4.5 (3.8–5.2)
South	21.4 (20.1–22.7)	17.5 (16.4–18.7)	14.8 (13.7–15.9)	3.8 (3.3–4.3)	1.0 (0.7–1.2)	3.5 (3.1–4.0)	2.9 (2.5–3.4)	3.9 (3.4–4.4)
West	15.3 (13.9–16.6)	12.7 (11.5–13.8)	10.7 (9.6–11.8)	2.6 (2.2–3.1)	1.1 (0.7–1.5)	2.9 (2.2–3.5)	1.7 (1.3–2.1)	3.0 (2.4–3.6)
**Education (adults aged ≥25 years)**								
0–12 yrs (no diploma)	25.9 (23.7–28.0)	23.1 (21.1–25.1)	21.8 (19.9–23.8)	2.8 (2.1–3.5)	—	2.5 (1.8–3.3)	2.9 (2.0–3.8)	4.2 (3.4–5.1)
GED	41.4 (36.2–46.7)	38.6 (33.5–43.8)	36.0 (31.3–40.7)	—	—	—	—	9.7 (6.9–12.4)
High school diploma	25.2 (23.6–26.9)	21.7 (20.1–23.2)	19.7 (18.3–21.1)	4.0 (3.3–4.7)	—	2.7 (2.2–3.3)	3.6 (2.9–4.2)	4.9 (4.0–5.7)
Some college, no degree	24.7 (23.0–26.3)	21.2 (19.6–22.8)	18.3 (16.7–19.8)	4.4 (3.7–5.2)	—	4.1 (3.3–4.9)	2.8 (2.2–3.4)	5.0 (4.2–5.8)
Associate degree	21.3 (19.6–23.1)	18.0 (16.4–19.6)	14.8 (13.3–16.3)	4.3 (3.4–5.2)	—	3.0 (2.3–3.6)	3.1 (2.3–3.8)	3.9 (3.0–4.8)
Undergraduate degree	13.0 (11.8–14.1)	10.6 (9.6–11.6)	7.1 (6.2–7.9)	3.7 (3.1–4.4)	1.1 (0.7–1.4)	2.2 (1.7–2.6)	1.5 (1.1–1.9)	2.0 (1.6–2.5)
Graduate degree	8.2 (7.1–9.4)	7.0 (5.9–8.0)	3.7 (3.0–4.4)	3.1 (2.4–3.8)	—	—	—	—
**Marital status**								
Married/Living with partner	18.4 (17.5–19.2)	15.3 (14.5–16.1)	12.5 (11.7–13.2)	3.7 (3.3–4.1)	0.8 (0.7–1.0)	2.6 (2.2–2.9)	2.6 (2.3–3.0)	3.3 (2.9–3.7)
Divorced/Separated/ Widowed	22.6 (21.2–24.0)	20.2 (19.0–21.4)	18.1 (16.9–19.4)	3.3 (2.7–3.8)	0.8 (0.5–1.1)	2.4 (2.0–2.9)	2.3 (1.8–2.8)	3.5 (3.0–4.0)
Single/Never married/Not living with a partner	21.1 (19.7–22.6)	17.2 (15.9–18.6)	13.9 (12.7–15.1)	4.8 (4.0–5.5)	1.7 (1.3–2.1)	5.5 (4.6–6.3)	1.7 (1.4–2.0)	4.9 (4.2–5.6)
**Income (USD)^¶¶¶^**								
<35,000	26.2 (24.8–27.6)	23.2 (22.0–24.5)	21.3 (20.0–22.5)	3.8 (3.3–4.3)	1.7 (1.3–2.1)	4.0 (3.4–4.5)	2.1 (1.7–2.6)	5.5 (4.8–6.1)
35,000–74,999	21.0 (19.8–22.3)	17.8 (16.7–19.0)	14.9 (13.8–16.0)	4.1 (3.5–4.7)	0.9 (0.7–1.2)	3.5 (2.9–4.0)	2.6 (2.1–3.1)	4.1 (3.6–4.7)
75,000–99,999	20.2 (18.5–21.9)	16.5 (15.0–18.1)	13.3 (11.8–14.8)	3.9 (3.1–4.6)	—	3.7 (2.8–4.6)	2.9 (2.2–3.6)	3.7 (2.8–4.5)
≥100,000	14.3 (13.1–15.5)	10.8 (9.8–11.8)	7.3 (6.5–8.2)	4.2 (3.5–4.8)	—	2.7 (2.2–3.3)	2.4 (1.9–2.9)	2.4 (1.9–2.8)
**Sexual orientation**								
Heterosexual/Straight	19.5 (18.8–20.3)	16.3 (15.7–17.0)	13.5 (12.9–14.1)	3.8 (3.5–4.2)	1.0 (0.8–1.1)	3.1 (2.8–3.4)	2.5 (2.2–2.7)	3.6 (3.3–4.0)
Lesbian, gay, or bisexual	29.2 (24.7–33.7)	24.9 (20.7–29.1)	20.6 (16.7–24.5)	—	—	—	—	—
Health insurance coverage****								
Private insurance	17.2 (16.4–18.0)	13.7 (13.0–14.4)	10.5 (9.9–11.1)	3.9 (3.5–4.3)	0.9 (0.7–1.1)	3.0 (2.7–3.4)	2.5 (2.2–2.8)	3.1 (2.7–3.4)
Medicaid	27.8 (25.6–30.0)	25.3 (23.2–27.5)	23.9 (21.8–26.0)	3.8 (3.0–4.5)	—	4.2 (3.2–5.1)	—	5.5 (4.5–6.5)
Medicare only (≥65 yrs)	12.6 (11.0–14.1)	10.9 (9.5–12.4)	9.4 (8.1–10.8)	—	—	—	—	—
Other public insurance	23.0 (20.5–25.5)	20.4 (17.9–22.8)	17.4 (15.1–19.8)	4.2 (3.2–5.3)	—	3.3 (2.3–4.3)	—	4.7 (3.5–5.9)
Uninsured	29.9 (27.4–32.4)	26.4 (24.1–28.8)	23.9 (21.7–26.1)	5.1 (4.0–6.2)	—	5.0 (3.9–6.1)	2.8 (2.0–3.7)	7.1 (5.9–8.4)
**Disability/Limitation^††††^**								
Yes	24.3 (22.4–26.3)	20.9 (19.0–22.7)	19.2 (17.3–21.0)	3.6 (2.7–4.4)	—	3.6 (2.9–4.4)	2.9 (2.1–3.7)	4.9 (4.0–5.9)
No	19.3 (18.5–20.0)	16.1 (15.4–16.7)	13.1 (12.5–13.7)	3.9 (3.6–4.3)	1.0 (0.9–1.2)	3.2 (2.9–3.5)	2.3 (2.1–2.6)	3.6 (3.3–3.9)
**Serious psychological distress^§§§§^**								
Yes	36.7 (32.7–40.6)	33.0 (29.0–37.0)	31.6 (27.9–35.4)	—	—	6.2 (4.6–7.8)	—	8.4 (6.2–10.6)
No	19.1 (18.4–19.8)	15.9 (15.2–16.5)	13.0 (12.4–13.6)	3.8 (3.5–4.2)	1.0 (0.9–1.2)	3.1 (2.8–3.4)	2.4 (2.1–2.6)	3.5 (3.2–3.8)

**Abbreviations:** AI/AN = American Indian/Alaska Native; CI = confidence interval; GED = General Educational Development certificate.

* Any tobacco product use was defined as use “every day” or “some days” of at least one tobacco product (for cigarettes, users were defined as persons who reported use either “every day” or “some days” and had smoked ≥100 times during their lifetime).

^†^ Any combustible tobacco product use was defined as use “every day” or “some days” of at least one combustible tobacco product: cigarettes; cigars, cigarillos, filtered little cigars; pipes, water pipes, or hookahs (for cigarettes, users were defined as persons who reported use either “every day” or “some days” and had smoked ≥100 times during their lifetime).

^§^ Current cigarette smokers were defined as persons who reported smoking ≥100 cigarettes during their lifetime and now smoked cigarettes “every day” or “some days.”

^¶^ Reported smoking cigars, cigarillos, or little filtered cigars at least once during their lifetime and now smoked at least one of these products “every day” or “some days.”

** Reported smoking tobacco in a regular pipe, water pipe, or hookahs at least once during their lifetime and now smoked at least one of these products “every day” or “some days.”

^††^ Reported using electronic cigarettes at least once during their lifetime and now used e-cigarettes “every day” or “some days.”

^§§^ Reported using chewing tobacco, snuff, dip, snus, or dissolvable tobacco at least once during their lifetime and now used at least one of these products “every day” or “some days.”

^¶¶^ Multiple tobacco product use was defined as use either “every day” or “some days” for at least two or more of the following tobacco products: cigarettes (≥100 times during lifetime); cigars, cigarillos, or filtered little cigars; pipes, water pipes, or hookahs; electronic cigarettes; or smokeless tobacco products.

*** Dashes indicate prevalence estimates with a relative standard error >30% that are not presented.

**^†††^** Hispanic persons could be of any race. All other racial/ethnic groups were non-Hispanic.

^§§§^ *Northeast:* Connecticut, Maine, Massachusetts, New Hampshire, New Jersey, New York, Pennsylvania, Rhode Island, and Vermont; *Midwest:* Illinois, Indiana, Iowa, Kansas, Michigan, Minnesota, Missouri, Nebraska, North Dakota, Ohio, South Dakota, and Wisconsin; *South:* Alabama, Arkansas, Delaware, District of Columbia, Florida, Georgia, Kentucky, Louisiana, Maryland, Mississippi, North Carolina, Oklahoma, South Carolina, Tennessee, Texas, Virginia, and West Virginia; *West:* Alaska, Arizona, California, Colorado, Hawaii, Idaho, Montana, Nevada, New Mexico, Oregon, Utah, Washington, and Wyoming.

^¶¶¶^ Based on income variables from the family file (n = 8,310 missing valid income data). Imputed income files were not used in this analysis.

**** *Private coverage*: includes adults who have any comprehensive private insurance plan (including health maintenance organizations and preferred provider organizations). *Medicaid*: for adults aged <65 years, includes adults who do not have private coverage, but who have Medicaid or other state-sponsored health plans including Children’s Health Insurance Program (CHIP); for adults aged ≥65 years, includes those who do not have any private coverage but have Medicare and Medicaid or other state-sponsored health plans including CHIP. *Medicare only*: includes adults aged ≥65 years who only have Medicare coverage. *Other coverage*: includes adults who do not have private insurance, Medicaid, or other public coverage, but who have any type of military coverage, coverage from other government programs, or Medicare. *Uninsured*: includes adults who have not indicated that they are covered at the time of the interview under private health insurance, Medicare, Medicaid, CHIP, a state-sponsored health plan, other government programs, or military coverage. Insurance coverage is “as of time of survey.”

^††††^ Disability was defined based on self-reported presence of selected limitations including vision, hearing, mobility, remembering, self-care, and communication. Respondents who answered “A lot of difficulty” or “Cannot do at all/unable to do” to one of the following questions “Do you have difficulty seeing, even when wearing glasses?,” “Do you have difficulty hearing, even when using a hearing aid?,” “Do you have any difficulty walking or climbing steps?,” “Using your usual language, do you have difficulty communicating, for example, understanding or being understood?,” “Do you have difficulty remembering or concentrating?,” “Do you have difficulty with self-care, such as washing all over or dressing?” to be coded as having a disability; those who responded “no difficulty” or “some difficulty” to all six questions were coded to not have a disability. These six questions are based on the short set of questions recommended by the Washington Group on Disability Statistics (https://www.cdc.gov/nchs/washington_group/index.htm).

^§§§§^ The Kessler psychological distress scale is a series of six questions that ask about feelings of sadness, nervousness, restlessness, worthlessness, and feeling like everything is an effort in the past 30 days. Participants were asked to respond on a Likert scale ranging from “None of the time” (score = 0) to “All of the time” (score = 4). Responses were summed over the six questions; persons with a score of ≥13 were coded as having serious psychological distress, and respondents with a score <13 were coded as not having serious psychological distress.

**FIGURE 1:**
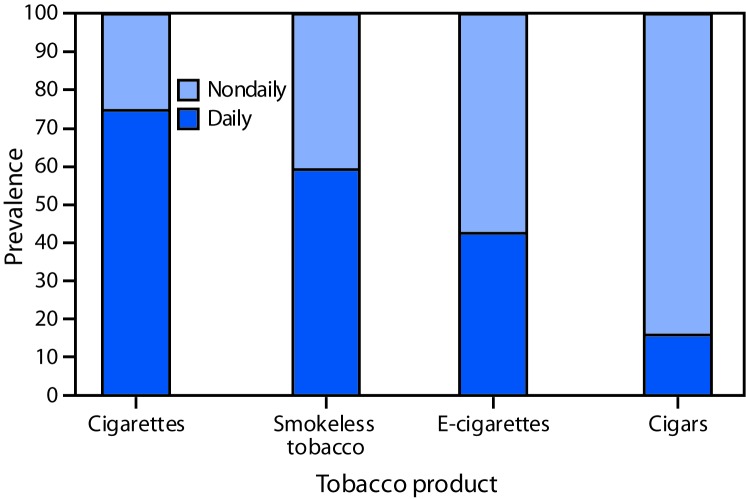
Prevalence of daily* and nondaily^†^ use of selected tobacco products^§^ among adults aged ≥18 years who currently use each tobacco product — National Health Interview Survey, United States, 2018 * Smoking cigarettes every day at the time of the survey among persons who reported having smoked ≥100 cigarettes during their lifetime or use of e-cigarettes, cigars, or smokeless tobacco every day at the time of survey. ^†^ Smoking cigarettes on some days at the time of survey among persons who reported having smoked ≥100 cigarettes during their lifetime or use of e-cigarettes, cigars, or smokeless tobacco on some days at the time of survey. ^§^ Daily use estimates for pipe use were unstable (relative standard error >30%; neither daily use nor nondaily use is presented.

The prevalence of any current tobacco product use was higher among males (25.8%) than among females (14.1%) and among persons aged 25–44 years (23.8%), 45–64 years (21.3%), and 18–24 years (17.1%) than among those aged ≥65 years (11.9%) (Table). Current tobacco product use was also higher among non-Hispanic American Indian/Alaska Native adults (32.3%), non-Hispanic multiracial adults (25.4%), non-Hispanic whites (21.9%), non-Hispanic blacks (19.3%), and Hispanic adults (13.8%) than among non-Hispanic Asian adults (10.0%), as well as among those who lived in the Midwest (23.6%) or the South U.S. Census regions (21.4%) than among those who lived in the West (15.3%) or the Northeast (17.5%). The prevalence of current tobacco product use was also higher among persons who had a GED (41.4%) than among those with other levels of education and among those who were divorced, separated, or widowed (22.6%) or single, never married, or not living with a partner (21.1%) than among those married or living with a partner (18.4%). Current tobacco product use was higher among persons with an annual household income <$35,000 (26.2%) than those in higher income groups, as well as among lesbian, gay, or bisexual adults (29.2%) than among those who were heterosexual (19.5%). Prevalence also was higher among adults who were uninsured (29.9%), insured by Medicaid (27.8%), or had some other public insurance (23.0%) than among those with private insurance (17.2%) or Medicare only (12.6%); among those who had a disability/limitation (24.3%); and those who had serious psychological distress (36.7%).

Significant linear increases occurred for all three cigarette cessation indicators. Among adult cigarette smokers, the prevalence of making a quit attempt in the past 12 months increased from 52.8% in 2009 to 55.1% in 2018 (p<0.001) (Figure 2). Recent successful smoking cessation increased from 6.3% in 2009 to 7.5% in 2018 (p<0.001). The quit ratio for cigarette smoking increased from 51.7% in 2009 to 61.7% in 2018 (p<0.001).

**FIGURE 2:**
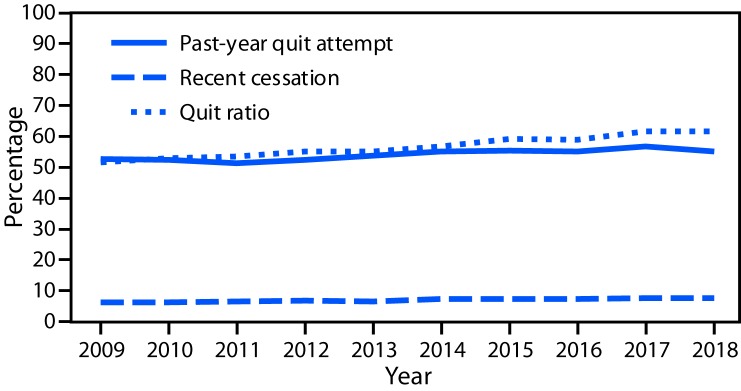
Prevalence of past-year quit attempts* and recent cessation^†^ and quit ratio^§^ among cigarette smokers aged ≥18 years — National Health Interview Survey, United States, 2009–2018 * Percentage of current cigarette smokers who reported they stopped smoking for >1 day during the past 12 months because they were trying to quit smoking and former smokers who quit during the past year. ^†^ Percentage of former cigarette smokers who quit smoking for ≥6 months during the past year, among current smokers who smoked for ≥2 years and former smokers who quit during the past year. ^§^ Percentage of persons who ever smoked (≥100 cigarettes during lifetime) who have quit smoking.

## Discussion

The approximate two thirds decline in adult cigarette smoking prevalence that has occurred since 1965 represents a major public health success (*1*). In 2018, 13.7% of U.S. adults aged ≥18 years currently smoked cigarettes, the lowest prevalence recorded since 1965. However, no significant change in cigarette smoking prevalence occurred during 2017–2018. Most cigarette smokers and smokeless tobacco users reported daily use, whereas most e-cigarette and cigar users reported nondaily use. Even nondaily use of cigarettes has been linked to increased mortality risk (*6*).

Quitting smoking at any age is beneficial for health (*1*,*4*). During 2009–2018, significant linear increases occurred in quit attempts, recent successful cessation, and quit ratio. Population-based tobacco control interventions, including high-impact tobacco education campaigns like CDC’s Tips From Former Smokers (https://www.cdc.gov/tobacco/campaign/tips/index.html) campaign and FDA’s Every Try Counts campaign (https://www.fda.gov/tobacco-products/every-try-counts-campaign), combined with barrier-free access to evidence-based cessation treatments, can both motivate persons who use tobacco products to try to quit and help them succeed in quitting.

The prevalence of adult e-cigarette use increased from 2.8% in 2017 to 3.2% in 2018 but was much lower than the 20.8% (*7*) of U.S. high school students reporting past 30-day e-cigarette use in 2018. The prevalence of e-cigarette use among persons aged 18–24 years is higher than that among other adult age groups, and e-cigarette use in this age group increased from 5.2% in 2017 (*2*) to 7.6% in 2018. During 2014–2017 there had been a downward trajectory of adult e-cigarette use (*2**,**8*), but during 2017–2018 a significant increase in adult e-cigarette use was detected for the first time. This increase might be related to the emergence of new types of e-cigarettes, especially “pod-mod” devices, which frequently use nicotine salts as opposed to the free-base nicotine used in other e-cigarettes and tobacco products. Sales of JUUL, a pod-mod device, increased by approximately 600% from 2016 to 2017, making it the dominant e-cigarette product in the United States by the end of 2017 (*9*). Further research is needed to monitor patterns of e-cigarette use and the relationship between use of e-cigarettes and other tobacco products (e.g., cigarette smoking).

The findings in this report are subject to at least three limitations. First, responses were self-reported and were not validated by biochemical testing. However, self-reported smoking status correlates highly with serum cotinine levels (*10*). Second, because NHIS is limited to the noninstitutionalized U.S. civilian population, the results are not generalizable to institutionalized populations and persons in the military. Finally, the NHIS Sample Adult response rate of 53.1% might have resulted in nonresponse bias.

Coordinated efforts at the local, state, and national levels are needed to continue progress toward reducing tobacco-related disease and death in the United States. Proven strategies include implementation of tobacco price increases, comprehensive smoke-free policies, high-impact antitobacco media campaigns, barrier-free cessation coverage, and comprehensive state tobacco control programs, combined with regulation of the manufacturing, marketing, and distribution of all tobacco products (*1*,*4*).

**Summary**

**What is already known about this topic?**

Cigarette smoking is the leading cause of preventable disease and death in the United States. Adult cigarette smoking prevalence has declined; however, new tobacco products, including e-cigarettes, have entered the U.S. market.

**What is added by this report?**

In 2018, approximately 20% of U.S. adults currently used any tobacco product; cigarette smoking reached an all-time low (13.7%). During 2009–2018, significant increases in three cigarette cessation indicators occurred. During 2017–2018, e-cigarette and smokeless tobacco product use prevalence increased.

**What are the implications for public health practice?**

Continued surveillance is critical to informing tobacco control efforts at the national, state, and local levels. Coordinated efforts and regulation of all tobacco products are needed to reduce tobacco-related disease and death in the United States.

## Footnotes

* The use of regular pipe, water pipe, or hookah was assessed together using a single question.

† https://www.cdc.gov/nchs/nhis/data-questionnaires-documentation.htm.

§ Use of two or more of the following tobacco products: cigarettes (≥100 cigarettes during lifetime); cigars, cigarillos, or filtered little cigars; pipes, water pipes, or hookahs; electronic cigarettes; or smokeless tobacco products every day or on some days.

¶ NHIS 2017 data were incorporated to inform statistically significant differences (p<0.05) during 2017–2018 for the use of any tobacco product, any combustible tobacco product, ≥2 tobacco products, cigarettes, cigars, pipes, e-cigarettes, and smokeless tobacco. The 2017 Sample Adult component included 26,742 adults aged ≥18 years; the response rate was 53.0%.

** Current cigarette smokers who reported they stopped smoking for >1 day during the past 12 months because they were trying to quit smoking and former smokers who quit during the past year.

†† Former cigarette smokers who quit smoking for ≥6 months during the past year, among current smokers who smoked for ≥2 years and former smokers who quit during the past year.

§§ The percentage of ever smokers (≥100 cigarettes during lifetime) who have quit smoking.

¶¶ Daily estimates of pipe use were statistically unstable (relative standard error>30%) and not presented.
